# Increased expression of MMP9 is correlated with poor prognosis of nasopharyngeal carcinoma

**DOI:** 10.1186/1471-2407-10-270

**Published:** 2010-06-09

**Authors:** Zhen Liu, Lixia Li, Zhixiong Yang, Weiren Luo, Xin Li, Huiling Yang, Kaitai Yao, Bin Wu, Weiyi Fang

**Affiliations:** 1 Cancer Research Institute, Key Lab for Transcriptomics and Proteomics of Human Fatal Diseases Supported by Ministry of Education and Guangdong Province, Southern Medical University, 510515,Guangzhou, PR China; 2 Department of Respiratory Medicine and Cancer Center, Affiliated Hospital of Guangdong Medical College, 524000, Zhanjiang, PR China; 3 Institute of Clinical Medicine, First Affiliated Hospital of University of South China, 421001, Hengyang, PR China

## Abstract

**Introduction:**

The aim of the present study was to analyze the expression of matrix metalloproteinase 9 (*MMP9*) in nasopharyngeal carcinoma (NPC) and its correlation with clinicopathologic features, including the survival of patients with NPC.

**Methods:**

Using real-time PCR, we detected the mRNA expression of *MMP9 *in normal nasopharyngeal tissues and nasopharyngeal carcinoma (NPC) tissues. Using immunohistochemistry analysis, we analyzed *MMP9 *protein expression in clinicopathologically characterized 164 NPC cases (116 male and 48 female) with age ranging from 17 to 80 years (median = 48.4 years) and 32 normal nasopharyngeal tissues. Cases with greater than or equal to 6 and less than 6 of the score value of cytoplasmic *MMP9 *immunostaining were regarded as high expression and low expression, respectively. The relationship between the expression levels of *MMP9 *and clinical features was analyzed.

**Results:**

The expression level of *MMP9 *mRNA was markedly greater in NPC tissues than that in the nasopharyngeal tissues. Immunohistochemical analysis revealed that the protein expression of *MMP9 *detected in NPC tissues was higher than that in the nasopharyngeal tissues (*P *= 0.004). In addition, high levels of *MMP9 *protein were positively correlated with the status of lymph node metastasis (N classification) (*P *= 0.002) and clinical stage (*P *< 0.001) of NPC patients. Patients with higher *MMP9 *expression had a significantly shorter overall survival time than did patients with low *MMP9 *expression. Multivariate analysis suggested that the level of *MMP9 *expression was an independent prognostic indicator (*P *= 0.008) for the survival of patients with NPC.

**Conclusion:**

High level of *MMP9 *expression is a potential unfavorable prognostic factor for patients with NPC.

## Background

Nasopharyngeal carcinoma (NPC) is one of the most common malignant diseases in the Chinese and other south-east Asians. Unfortunately, most NPC patients tend to present a more advanced stage of disease when first diagnosed due to its deep location and vague symptoms. Therefore, it is of great interest to search valuable factors for early diagnosis, prognosis prediction, and novel therapeutic strategies.

The formation and metastasis of NPC is a complex and continuous process with the participation of a number of key genes [1]. In a previous study, we used cDNA microarray to detect differentially expressed genes among NPC tissues and non-cancerous nasopharyngeal tissues. By means of the analysis of BRB-array tools, the expression of *MMP9*, a gene encoding matrix metalloproteinase 9, was shown to be markedly upregulated in NPC tissues, suggesting a possible role of *MMP9 *in promoting the pathogenesis of NPC [2].

*MMP9*, a member of the matrix metalloproteinases (*MMPs*), plays a critical role in breakdown of extracellular matrix in normal physiological processes, such as embryonic development, reproduction, and tissue remodeling, as well as in disease processes, such as tumor metastasis[3]. *MMP9 *is secreted from cells and, once activated, is thought to degrade collagen in the extracellular matrix, which promotes the metastasis of tumor cells [4].

In order to clarify the role of *MMP9 *in the pathogenesis of NPC, in the present study we investigated the correlation of *MMP9 *protein expression with clinicopathologic features, including the survival of patients. We found that the mRNA and protein expression levels of *MMP9 *were higher in NPC tissues than those in non-cancerous nasopharyngeal tissues. Furthermore, the relatively higher protein expression of *MMP9 *was associated with NPC progression and poor prognosis. Our results suggest that overexpressed *MMP9 *is an unfavorable prognostic factor for NPC patient's survival.

## Materials and methods

### Sample collection

Seven primary fresh NPC samples and 5 non-cancerous fresh nasopharyngeal samples were collected from the People's Hospital of Zhongshan City, China, at the time of diagnosis before any therapy. All fresh samples were immediately preserved in liquid nitrogen. One hundred and sixty four undifferentiated NPC specimens and 32 non-cancerous nasopharyngeal specimens, both paraffin-embedded, were obtained from the People's Hospital of Zhongshan City and the First Affiliated Hospital of Guangdong Medical School, Zhanjiang City, China. In the 164 NPC cases, there were 116 male and 48 female with age ranging from 17 to 80 years (median, 48.4 years). For the use of these clinical materials for research purposes, prior consents from the patients and approval from the Ethics Committees of these two hospitals were obtained. All specimens had confirmed pathological diagnosis and were staged according to the 1997 NPC staging system of the WHO.

### Real-time PCR

Real-time PCR was performed to measure the expression of *MMP9 *mRNA in 7 fresh NPC tissues and 5 fresh nasopharyngeal tissues using SYBR Premix Ex Taq (Takara, Japan) with an Mx3000P real-time PCR system (Stratagene, La Jolla, CA, USA) as described previously [5]. The sequence for sense primer was 5'-GAGTGGCAGGGGGAAGATGC-3', and for antisense primer was 5'-CCTCAGGGCACTGCAGGATG-3'. *GAPDH *gene was used as an internal control using the sense primer 5'-GCACCGTCAAGGCTGAGAAC-3' and antisense primer 5'-TGGTGAAGACGCCAGTGGA-3'.

### Immunohistochemistry

Paraffin sections (3 μm) from samples of 164 NPC and 32 nasopharyngeal specimens were deparaffinized in 100% xylene and re-hydrated in descending ethanol series(100%, 90%,80%, 70% ethanol) and water according to standard protocols. Heat-induced antigen retrieval was performed in 10 mM citrate buffer for 2 min at 100°C. Endogenous peroxidase activity and non-specific antigen were blocked with peroxidase blocking reagent containing 3% hydrogen peroxide and serum, followed by incubation with goat anti-human *MMP9 *antibody (1:100) (Abcam, MA, USA) for 1 h at 37°C. After washing, the sections were incubated with biotin-labeled rabbit anti-goat antibody for 10 min at room temperature, and subsequently were incubated with streptavidin-conjugated horseradish peroxidase (HRP) (Maixin Inc, China). The peroxidase reaction was developed using 3, 3-diaminobenzidine chromogen solution in DAB buffer substrate. Sections were visualized with DAB and counterstained with hematoxylin, mounted in neutral gum, and analyzed using a bright field microscope.

### Evaluation of staining

The immunohistochemically stained tissue sections were reviewed and scored separately by two pathologists blinded to the clinical parameters. The staining intensity was scored as previously described [6,7]. The extent of the staining, defined as the percentage of positive staining areas of tumor cells or normal nasopharyngeal epithelial cells in relation to the whole tissue area, was scored on a scale of 0 to 4 as the following: 0, < 10%; 1, 10-25%; 2, 26-50%; 3, 50-75%; and 4, >76%. The sum of the staining-intensity and staining-extent scores was used as the final staining score for *MMP9 *(0-7). For statistical analysis, a final staining scores of 0-5 and 6-7 were respectively considered to be low and high expression.

### Statistical analyses

All statistical analyses were performed using SPSS 13.0 software. Data were presented as mean ± SD. The χ^2 ^test was used to analyze the relationship between the levels of *MMP9 *expression and clinicopathologic characteristics. Survival curves were plotted using the Kaplan-Meier method and compared using the log-rank test. The significances of various variables in survival were analyzed using multivariate cox proportional hazards model. A *P *value of less than 0.05 was considered statistically significant.

## Results

### *MMP9 *mRNA was highly expressed in NPC tissue

**Figure 1:**
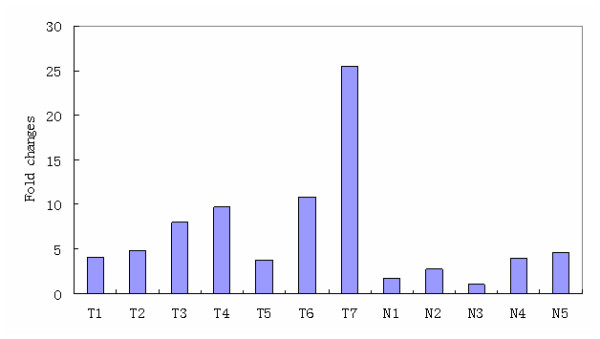
**Expression detection of *MMP9 *mRNA in NPCs and NPs**. MMP9 mRNA was highly expressed in NPC tissues compared with NP tissues (3.4 folds).**T**:Tumor; **N**:Normal.

In order to assess the role of *MMP9 *in NPC, we performed real-time PCR to measure the expression of *MMP9 *mRNA transcripts in 7 freshly collected NPC tissues and 5 freshly collected normal nasopharyngeal tissues. Compared with normal nasopharyngeal tissues, NPC tissues showed higher expression levels of *MMP9 *mRNA with an average increase of 3.4-fold (Figure 1).

### Immunohistochemical analysis of *MMP9 *protein expression in NPC and nasopharyngeal tissues

**Figure 2:**
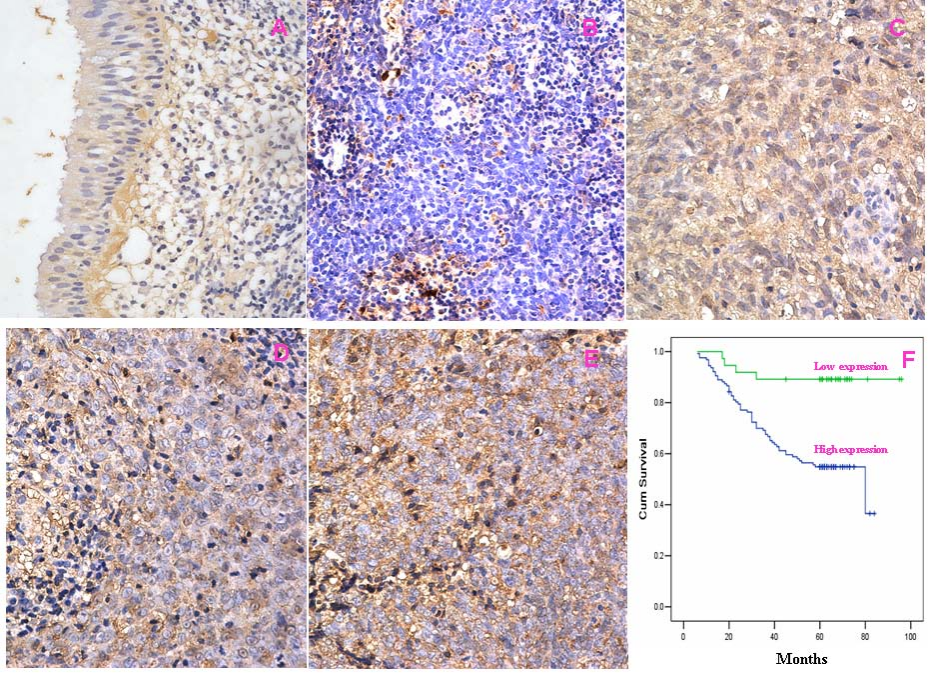
**Kaplan-Meier plots of overall survival duration in patients with NPC**. MMP9 protein expression in NPC and NP samples(A-E): **A**:Weak expression of MMP9 in NP sample; **B**:Negative expression of MMP9 in NPC samples(original magnification 400×);**C-E**:Strong staining of MMP9 in NPC samples (original magnification 400×). **F**. Kaplan-Meier survival analysis of overall survival duration in 164 NPC patients according to MMP9 protein expression. The log-rank test was used to calculate *p *values.

We measured the expression levels and subcellular localization of *MMP9 *protein in 164 archived paraffin-embedded NPC samples and 32 non-cancerous nasopharyngeal samples using immunohistochemical staining (Figure 2A-E). Specific *MMP9 *protein staining was found in the cytoplasm of non-cancerous and malignant epithelial cells. Furthermore, we observed that in 77.4% (127/164) of NPC samples, *MMP9 *protein was highly expressed. In comparison, only 53.1% of non-cancerous nasopharyngeal samples had highly expressed *MMP9 *protein, significantly lower than that in the NPC samples (*P *= 0.008) (Table 1).

**Table 1:** Protein expression of MMP9 between NPC and nasopharyngeal samples

Group		Protein expression		P value
	Cases	High expression	Low expression	
Cancer	164	127(77.4)	37 (22.6)	
Normal	32	17(53.1)	15 (46.9)	0.008

### Relationship between clinicopathological characteristics and *MMP9 *expression in NPC patients

The relationships between clinicopathological characteristics and *MMP9 *expression levels in individuals with NPC are summarized in Table 2. We did not find a significant association of *MMP9 *expression levels with patient's age, sex, smoking, tumor size (T classification), and status of distant metastasis (M classification) in 164 NPC cases. However, we observed that the expression level of *MMP9 *was positively correlated with the status of lymph node metastasis (N classification) (N0-N1 *vs*. N2-N3) (*P *= 0.002), and clinical stage (I-II *vs*. III-IV) (*P *< 0.000) in NPC patients (Table 3).

**Table 2:** Clinicopathologic characteristics of patient samples and expression of MMP9 in NPC

	N (%)
Gender	
Male	116 (70.7)
Female	48 (29.3)
Age (y)	
<50	83 (50.6)
≥50	81 (49.4)
Smoking	
Yes	39 (23.8)
No	125 (76.2)
T classification	
T_1_-T_2_	116 (70.3)
T_3_-T_4_	48 (29.3)
N classification	
N_0_-N1	74 (45.1)
N_2_-N_3_	90 (54.9)
Distant metastasis	
Yes	8 (5.1)
No	156 (94.9)
Clinical stage	
I~II	55 (33.5)
III~IV	109 (66.50)
Status at follow-up	
Alive	102 (62.2)
Death secondary to NPC	62 (38.8)
Expression of MMP9	
High expression	127 (77.4)
Low expression	37 (22.6)

**Table 3:** Correlation between the clinicopathologic characteristics and expression of MMP9 protein in NPC

		MMP9 (%)		
			
Characteristics	n	High expression	Low expression	*P*
Gender				
Male	116	94(81.0)	22 (19.0)	
Female	48	33(43.6)	15 (56.4)	0.102
Age( y)				
≥50	81	61 (75.3)	20 (24.7)	
<50	83	66 (47.7)	17(52.3)	0.577
Smoking				
Yes	39	31 (79.5)	8 (20.5)	
No	125	96 (76.8)	29 (23.2)	0.828
T classification				
T_1_-T_2_	116	85 (73.3)	31(26.7)	
T_3_-T_4_	48	42 (87.5)	6(12.5)	**0.064**
N classification				
N_0_-N_1_	74	49 (66.2)	25 (33.8)	
N_2_-N_3_	90	78 (86.7)	12 (13.3)	**0.002**
Distant metastasis				
Yes	8	7 (87.5)	1 (12.5)	
No	156	120 (76.9)	36 (23.1)	**0.685**
Clinical stage				
I~II	55	30 (54.5)	25 (45.5)	
III~IV	109	97 (89)	12(11)	**0.000**

### Survival analysis

To investigate the prognostic value of *MMP9 *expression for NPC, we assessed the association between the levels of *MMP9 *expression and patients' survival using Kaplan-Meier analysis with the log-rank test. In 164 NPC cases with prognosis information, we observed that the level of *MMP9 *protein expression was significantly correlated with the overall survival of NPC patients (Figure 2F). Patients with high level of *MMP9 *expression had poorer survival than those with lower level of *MMP9 *expression (*P *= 0.001). In addition, T, N, M classifications and clinical stages were also significantly correlated with patients' survival (*P *= 0.034, *P *< 0.001, *P *< 0.001, and *P *< 0.001 respectively). To determine whether *MMP9 *is an independent prognostic factor for NPC, we performed multivariate analysis of the levels of *MMP9 *protein expression adjusted for age, gender, smoking status, T classification, N classification, M classification, and clinical stages of NPC patients. The results showed that the level of *MMP9 *expression was an independent prognostic factor for NPC (Table 4).

**Table 4:** Summary of univariate and multivariate Cox regression analysis of overall survival duration

	Univariate analysis			Multivariate analysis		
		
Parameter	*P*	HR	95%CI	*P*	HR	95%CI
Age						
≥50 vs. <50 years	0.110	0.664	0.402-1.097	0.038	0.556	0.320-0.967
Gender						
Male vs. female	0.812	1.068	0.621-1.836	0.868	1.050	0.588-1.875
Smoking						
Yes vs. No	0.538	0.836	0.473-1.477	0.915	0.966	0.511-1.826
T classification						
T_1_-T_2 _vs. T_3_-T_4_	0.034	1.739	1.042-2.901	0.191	1.519	0.812-2.840
N classification						
N_0_-N1 vs. N_2--_N_3_	0.000	2.943	1.664-5.205	0.018	2.849	1.197-6.782
M classification						
M_0 _vs. M_1_	0.000	0.218	0.093-0.509	0.002	0.242	0.097-0.607
Clinical stage						
I-II vs. III-IV	0.000	4.323	2.055-9.092	0.971	1.021	0.321-3.251
MMP expression						
High expression **vs**. Low expression*	0.001	5.293	1.918-14.603	0.003	5.193	1.728-15.608

## Discussion

NPC is a malignant neoplasm arising from the mucosal epithelium of the nasopharynx, most often within the lateral nasopharyngeal recess and has been thought to be closely associated with Epstein-Barr virus infection, dietary, and genetic factors. The majority of the NPC deaths attribute to tumor metastases rather than primary tumors. However, the molecular mechanism of NPC invasion and metastasis remains incompletely understood.

In a recent microarray analysis, we found the significantly elevated level of *MMP9 *mRNA in NPC compared to non-cancerous nasopharyngeal tissues [2]. In this report, we found that *MMP9 *was expressed predominantly in the epithelial cells in both NPC and non-cancerous nasopharyngeal tissues by immunohistochemistry assay, which was consistent with Horikawa's result[8]. Similar to a report from Wong et al [9], we further presented the evidence that *MMP9 *was overexpressed at both mRNA and protein levels in NPC tissues compared to nasopharyngeal tissues, suggesting that *MMP9 *was involved in the pathogenesis of NPC.

*MMP9 *is a Zn^2+ ^dependent endopeptidase that mediates the degradation of extracellular matrix protein [10], and is associated with tumor invasion and metastasis [11,12]. It is synthesized and secreted in monomeric form as zymogen, and belongs to the gelatinase subgroup. Increased expression of *MMP9 *is usually seen in invasive and metastatic cancers such as colorectal cancer [13], gastric carcinoma [14], pancreatic carcinoma [15], breast cancer [16], and oral cancer [17]. The levels of *MMP9 *expression have also been found to be increased in nasal NK/T-cell lymphoma [18], malignant astrocytomas, carcinomatous meningitis, and brain metastases [19]. In this study, we also found that *MMP9 *overexpression was significantly associated with T classification (tumor size), N classification (lymph node metastasis), and clinical stages of NPC patients. Overexpressed *MMP9 *in NPC may accelerate tumor growth by inducing angiogenesis and enhance local cell invasion and metastasis by degrading the extracellular matrix. Our results may indicate that *MMP9 *plays significant roles in NPC progression, including tumor invasion and metastasis. Similarly, Horikawa et al also showed that overexpressed *MMP9 *protein was positively correlated with lymph node metastasis of NPC [8]. Furthermore, the expression of *MMP9 *also showed a significant positive correlation with the expression of oncoprotein *LMP1 *in NPC tissues. Transfection of a *LMP1 *expression plasmid into C33A cell line could increase *MMP9 *expression [20]. These studies consistently suggest that overexpressed *MMP9 *may play an unfavorable role in NPC pathogenesis. However, the correlation between *MMP9 *expression and the survival of NPC patients has been seldom reported.

In the past few years, *MMP9 *overexpression in tumor cells has been shown to be an independent prognostic factor in several types of tumors, which has a favorable or unfavorable prognostic significance depending on tumor types[21-24]. In epithelial ovarian cancer, the higher the amount of *MMP9 *positive cancer cells, the longer was the 10-year disease-related survival (DRS) [21]. Interestingly, similar results were reported in breast cancer. Pellikainen found that in breast cancer, positive *MMP9 *expression in cancer cells favored patient's survival [22]. On the contrary, there was more evidence indicating that overexpression of *MMP9 *in cancer cells was not a favorable prognosis factor in non-small cell lung cancer(NSCLC)[23], colorectal cancer[24], and oesophageal carcinoma, etc[25]. In NSCLC and colorectal cancer, overexpressed *MMP9 *was markedly associated with shortened cancer-related survival. In oesophageal carcinoma, *MMP9 *overexpression was significantly correlated with the depth of tumor invasion, lymphatic permeation, nodal metastasis, and pathologic differentiation grade.

In the present study, we presented the evidence that *MMP9 *protein expression in NPC was inversely correlated with patient's overall survival. The patients with higher expression of *MMP9 *protein had shorter survival time. According to multivariate analyses, increased expression of *MMP9 *protein was a significant predictor of poor prognosis for NPC patients, especially for its patients at late-stage. These results were analogous to Li et al's report[26] but inconsistent with Wong et al's investigation[9]. Li et al used immunohistochemistry assay in NPC tissues to find that NPC patients with dual high-expression of *MMP9/PAR-2 *showed a significantly worse prognosis than those with single highly expressed protein or dual low or negatively expressed proteins, which strongly supported the reliability of our study. However, Wong et al reported that the increased level of plasma *pro-MMP9 *by enzyme linked immunosorbant assay did not correlate with NPC patients' clinical outcome. The discrepancy between our data and Wong et al's data would be most likely due to the different samples and method used.

## Conclusion

In summary, our study demonstrated that the expression level of *MMP9 *was significantly increased in NPC and correlated with the malignant status of NPC. Furthermore, our data suggested that *MMP9 *was an important prognostic factor for NPC. Yet, due to the limited sample size of patients in our investigation, further studies would be needed to verify these findings and establish the role of *MMP9 *as a reliable clinical predictor for the outcome of NPC patients.

## Disclosure of Potential Conflicts of Interest

No potential conflicts of interest were disclosed

## Authors' contributions

Z.L., B.W., L.L., W.L., X.L. and H.Y. performed this research. Z.L. and W.F. collected, analyzed, and interpreted data and wrote the manuscript. Z.Y. collected and analyzed data. W.F., Z.L., K.Y. and B.W. supervised all the work. All authors have read and approved the final manuscript.

## Pre-publication history

The pre-publication history for this paper can be accessed here:

http://www.biomedcentral.com/1471-2407/10/270/prepub
